# Sulphate of Quinine in Aneurism of the Aorta, and in Other Internal Anuerisms

**Published:** 1847-10

**Authors:** 


*Sulphate of Quinine in Aneurism of the Aorta, and in other internal Anuerisms.*—It appears that sulphate of quinine has been employed with much success in some Italian hospitals for the relief of aneurism of the aorta and other internal aneurisms. It belongs, in this use of it, to what are termed hyposthenics, (subduing action,) and is to be carried as far as the system will bear it. It has, says its Italian supporters, the immense advantage of bringing down the pulse without disturbing its rhythm, of making the buffy coat of the blood disappear, that is, of dissipating the organic condition,—namely, arteritis, on which it depends, and thus of retarding the progress of the aneurismal tumour. The other hyposthenics adapted to the same end according to the same authorities, as by alternation with the sulphate of quinine, are the vegetable and mineral acids, the sulphate of iron, the ergot of rye, the cold ferruginous waters, the arsenious acid, the acetate of lead, and the iodide of potassium.—*Ibid.*

